# The Janus Structure of Graphene Oxide and Its Large-Size Conductive Film Strip Pattern

**DOI:** 10.3390/nano14110980

**Published:** 2024-06-05

**Authors:** Lu Yi, Xiangnan Chen, Heng Su, Chaocan Zhang

**Affiliations:** School of Materials Science and Engineering, Wuhan University of Technology, Wuhan 430070, China; 317309@whut.edu.cn (L.Y.); chenxiangnan5523@163.com (X.C.); suhengwhut@163.com (H.S.)

**Keywords:** graphene, graphene oxide, janus structures, self-printing circuits, conductivity

## Abstract

In this paper, the oxidation–exfoliation process of graphite is studied experimentally by the mixed-solvent method, the oxidation–exfoliation process of graphite is simulated theoretically, and it is found that Graphene Oxide (GO) is a Janus structure with inconsistent oxidation on both surfaces; hydrophilic on one side and hydrophobic on the other side. This layer structure and layer spacing are due to the inconsistent oxidation on both sides which changes with the polarity of different solvent mixtures. We used a two-phase system of benzyl alcohol and water, as well as controlling the polarity of the surface of the substrate, to achieve (using a mixed solution of GO which has a selectivity more inclined to the oil phase when the aqueous phase is present) the preparation of reduced graphene oxide patterns. We also used a complex solution of hydrogen iodide and a sodium–iodide complex solution for secondary reduction to enhance its conductivity to 8653 S/m.

## 1. Introduction

Graphene is a two-dimensional carbon nanomaterial with a hexagonal honeycomb lattice consisting of carbon atoms in sp2 hybridisation orbitals [1]. Graphene has been widely studied for its unique physicochemical properties such as its large specific surface area [2], good electrical conductivity [3], high electron mobility, and high mechanical strength [4], and, in recent years, it has become a material that has attracted a lot of attention in the field of electronics [5,6].

Typically, graphene can be prepared by physical methods such as exfoliation [7,8,9,10,11,12] and chemical methods such as redox [13,14,15]. The redox method [16,17,18,19,20] is based on graphite, which is oxidised to produce graphene oxide (GO) and then reduced to obtain it. It is one of the most important preparation methods due to the advantages of a simple process, easy control of conditions, low cost, and scalable production. The more commonly used methods for the preparation of GO are Brodie’s method [21], Staudenmaier’s method [22], and Hummers’ method [23], etc. GO is mainly prepared by the potassium permanganate (KMnO_4_) system from Hummers’ method. Depending on the reaction conditions, the degree of oxidation of graphite changes with them, and the C/O ratios of the prepared GOs are mostly in the range of 1.33–3 [24,25,26,27,28,29]. According to what has been reported in the literature, GO has a sharp peak in the XRD spectrum, and the position of the peak deviates due to the different reaction conditions, usually within 8.7°–10.2° [30,31,32], suggesting that it is a layered stacked structure with a layer spacing of 8.66–10.15 Å. The structure of GO has been studied across the literature, and it has been found that the types and contents of its oxidation functional groups are complex and diverse [33,34], with hydroxyl and epoxy groups being the main oxidation functional groups on the GO carbon ring, while other functional groups such as carboxyl and carbonyl groups are mainly distributed at the edges [35,36,37,38]. Stankovich et al. [39] proposed that these oxygen functional groups in graphite oxide alter the van der Waals interactions between the graphite oxide layers, making them hydrophilic. The presence of these oxygen-containing functional groups makes GO readily soluble in strong polar solvents such as water [40,41,42,43,44,45] and N-methylpyrrolidone (NMP) [46,47,48,49], and insoluble in weakly polar solutions such as hexane and chloroform [50,51]. A series of theoretical models have been proposed for describing the structure of GO molecules, including the Hofmann model [52], the Ruess model [53], the Scholz–Boehm model [54], the Nakajima–Matsuo model [55], the Lerf–Klinowski model [35], and the Dékány model [37]; all of these models further validate the species and distribution of GO functional groups.

Interest in patterned, conductive, and flexible graphene electrodes has surged due to the multiple uses of graphene in flexible and wearable electronics. Various methods have been reported for patterning on flexible surfaces using graphene, such as the Langmuir Blodgett (LB) method, spin-coating [56], layer-by-layer assembly, and ink-jet printing. Among these, layer-by-layer assembly is a common method for depositing layered materials onto a substrate. The use of multiple interactions (electrostatic interactions, hydrogen bonding, covalent bonding, etc.) to drive the self-assembly of the deposited layers on the substrate can reduce the deposition cycle. The literature reports the use of GO, its assembly into different substrate materials, and then reduction to obtain conductive films. Zhao et al. [57] prepared conductive films by self-assembling GO at the liquid phase–gas phase interface, followed by fishing it out with polyethylene terephthalate (PET), drying it, and then allowing GO to be deposited, and the films had a square resistance of 840 Ω/sq at 78% light transmittance. Liu et al. [58] mixed GO aqueous solution with hydrazine hydrate and then diluted it with water/ethanol. A uniform and stable GO–hydrazine dispersion was obtained by ultrasound, which was stored for different times, and the rGO film was prepared by the direct spraying of the GO–hydrazine dispersion without a subsequent reduction process. The obtained transmission rate was 75% and the block resistance was 3.4 kΩ/sq.

Based on what has been reported in the literature, as well as our previous work [59,60], this has been shown to be a regular laminated stacked structure. In general, if the two sides of GO are uniformly oxidised, this will destroy the symmetry of the ring structure and it becomes difficult to have a layered structure, so we presume that GO is an asymmetric oxidised structure. In this paper, through experimental studies as well as the mixed solvent method, and using theoretical simulations of the oxidation–exfoliation process of graphite, it has been found that GO is a Janus structure with inconsistent oxidation on both sides, one hydrophilic and the other hydrophobic. This lamellar structure and spacing of layers, due to inconsistent oxidation on both sides, changes with the polarity of the different solvent mixtures. Based on the above findings, we used a two-phase system of benzyl alcohol and water, as well as controlling the polarity of the substrate surface when the mixed solution of GO has a selectivity more inclined to the oil phase in the presence of the aqueous phase, to achieve the preparation of reduced graphene oxide patterns, which can be used for self-printed circuits, to greatly enhance their conductivity by secondary reduction.

## 2. Experimental Methods

### 2.1. Materials

Natural crystalline flake graphite (NG) (99.85% purity, 325 mesh), sulfuric acid (H_2_SO_4_, 98%), potassium permanganate (KMnO_4_), hydrogen peroxide (H_2_O_2_, 30%), hydrochloric acid (HCl, 36%), 2-methoxy ethanol (EGM, 99%), and anhydrous ethanol (C_2_H_6_O ≥ 99.7%) were purchased from Sinopharm Chemical Reagent Co., Ltd. (Shanghai, China). Benzyl alcohol (BnOH, 99.8%), Hydrazine hydrate solution (99.8%), N,N-Dimethylformamide (DMF, 99.5%), Dimethyl sulfoxide (DMSO, 99.5%), 1-Methyl-2-pyrrolidinone (NMP), Ethylene glycol monophenyl ether (EPH), Hydriodic acid (HI, 57wt.%), Sodium iodide (NaI, 99.99%), Chitosan, Xylene (DMB), and Diethy phthalate (DEP) were obtained from Aladdin. OP-7 and (3-Aminopropyl)triethoxysilane (KH550) were purchased from Wen Hua Chemical Reagent Factory (Tianjin, China).

### 2.2. Preparation of Graphene Oxide

Concentrated H_2_SO_4_ and deionised water in different volume ratios were added to a 250 mL three-necked flask at room temperature, and 1 g of graphite powder was poured in and stirred for 30 min in an ultrasonically sound environment to disperse the powder uniformly. The three-necked flask was placed in an ice bath with continuous stirring, 5 g of KMnO_4_ was added slowly, and the temperature of the system was controlled to be less than 5 °C at this time. After the addition, the system was moved to room temperature and stirred for another 30 min to fully react the KMnO_4_. After 30 min, the system was moved to a water bath at 51–52 °C with continuous stirring for more than 12 h. The water bath was removed, and 100 mL of deionised water was slowly added dropwise with a constant pressure funnel, controlling the system temperature not to exceed 60 °C. The system was then stirred for 10 min at room temperature and then stirred for 10 min at room temperature. After the dropwise addition, the system was stirred for another 10 min, and then H_2_O_2_ was added slowly until no more bubbles were produced. The final product was dispersed by centrifugation, hydrochloric acid washing, centrifugation, and sonication for 4–5 h. Concentrated H_2_SO_4_ and deionised water were added in the ratios shown in Table 1 below.

### 2.3. janus-GO Experimental Tests

Take 10 mL of GO/EGM solution at a concentration of 5 mg/mL, add 10 mL of the following solvents dropwise while stirring: N,N-dimethylformamide (DMF), dimethylsulfoxide (DMSO), N-methylpyrrolidone (NMP), ethylene glycol phenyl ether (EPH), OP-7, xylene, and then ultrasonic for two hours, and then take the appropriate amount of the solution and put it on the surface of a dish, and then dry it under vacuum for 72 h at 50 °C. The dried sample was then subjected to an X-ray diffraction test. After two hours of sonication, an appropriate amount of the solution was placed in a surface dish and dried under vacuum at 50 °C for 72 h, and the dried sample was subjected to an X-ray diffraction test, and the GO/EGM solution, without other solvents, was used as the control sample, which was also dried and tested.

An amount of 10 mg of GO was added to 0.5 mL, 1 mL, and 2 mL of hydrophobic DEP, while 10 mg of GO was added to 20 mg and 50 mg of hydrophilic chitosan, respectively, and ultrasonication was carried out for two hours, and then an appropriate amount of the sample was placed in a surface dish, and the dried sample was vacuum dried for 48 h at 50 °C. The dried samples were subjected to an X-ray diffraction test.

### 2.4. MS Simulation of janus-GO

The oxidation–exfoliation process of graphite was simulated using Materials Studio (MS) to calculate the interaction energy between graphite oxide flakes and graphite flakes with different degrees of oxidation in sulphuric acid (H_2_SO_4_), in order to obtain the degree of oxidation at which the graphite oxide flakes start to exfoliate. The binding energy of the whole in H_2_SO_4_ is calculated and denoted as *E_T_*. The interaction energy of individual graphite flakes (*E*_*S*1_) and the interaction energy of individual graphite flakes with different degrees of oxidation (*E*_*S*2_) are then calculated. The action energy of exfoliation between graphite flakes and graphite of different oxidisation degrees can be calculated using the following formula:

$$E=E_{T}-E_{S1}-E_{S2}$$

Meanwhile, the interaction energies of the janus-GO hydrophilic and hydrophilic surfaces, the hydrophobic and hydrophobic surfaces, and the hydrophilic and hydrophobic surfaces when they are in H_2_O, BnOH, and DEP, respectively, are calculated using MS. In this case, a monolayer janus-GO molecular model was constructed with a C/O ratio consistent with the XPS results of the fabricated GO. Then, the interaction energy of the whole in different solvents was calculated and denoted as *E_T_*. Then, the interaction energy of a single janus-GO sheet (*E_SA_*_(*B*)_) was calculated. The interaction energy of janus-GO in different solvents can be calculated by the following equation:

$$E=E_{T}-E_{SA\left(B\right)}-E_{SA\left(B\right)}$$

### 2.5. Preparation of Conductive Films

Glass slice treatment: Firstly, the glass sheets were soaked in piranha solution for 48 h and washed with water and set aside. Then, the surface of the glass sheet was coated with a layer of mixed solvent in the ratio of anhydrous ethanol/DI water/KH550 = 9:1:0.02 and placed in an oven at 60 °C for the drying process. After the surface of the glass sheet was completely dry, it was removed and allowed to cool for subsequent use.

Preparation of conductive films: 5.75 mL of GO/EGM solution (40 mg of GO) was added to 20 mL of benzyl alcohol and sonicated for two hours to completely disperse the GO, and was ready to use. Place the glass slice in a Petri dish and pour the slurry in until it covers the glass surface. An equal volume of deionised water was added in slow drops along the edge of the Petri dish wall. Finally, continuous heating using an infrared searchlight was applied for about 4 to 5 h to promote the reduction of the graphene oxide. In this paper, two reducing agents, phenylhydrazine and hydrazine hydrate, were used, respectively: one so that 2 mL of phenylhydrazine was dissolved in the oil phase of GO/BnOH solution; the other so that 2 mL of 1.54 mol/L hydrazine hydrate was dissolved in the aqueous phase.

Preparation of reduced graphene oxide patterns: The difference in this preparation method lies in the treatment of the glass surface, in the use of KH550 mixed solvent, the use of brushes dipped in a small amount of mixed solvent, writing the mark “Whut” on the glass sheet, the drying and then use, the subsequent process and the preparation of conductive film is the same as the preparation method.

### 2.6. Secondary Reduced Graphene Films

The above prepared conductive films were placed in three different ratios of the reductant for the second reduction; the reductant type and the actual dosage are displayed in Table 2; the reaction temperature was 130 °C; the reaction time was 6 h. At the end of the reaction, the films were placed into a vacuum oven at 120 °C overnight.

### 2.7. Characterization of Conductive Film

The product moieties were analysed using a Nicolet/is 50 infrared spectrometer from Thermo Fisher Scientific, Waltham, MA, USA. The product defects were analysed using a TriStar II 3020 laser Raman spectrometer from Mack Instruments, Atlanta, GA, USA. The structure of the products was analysed using a D8-Advance X-ray diffractometer from Bruker, Saarbrücken, Germany. The C and O contents of the products were analysed using the ESCALAB 250 Xi X-ray photoelectron spectrometer from Thermo Fisher Scientific, Waltham, MA, USA. The surface morphology of the products was analysed using a JSM-IT300’ scanning electron microscope from Zeiss Optical Instruments, Oberkoche, Germany. The conductivity of the product was analysed by the multi-function digital four-probe tester (FT-341) of Ningbo Ruike Micro Intelligent Technology Co., Ltd., Ningbo, China. The specific model of the multi-function digital four-probe tester is shown in support information Figure S4 and Table S1.

## 3. Results and Discussion

### 3.1. GO Preparation and Characterisation Studies

#### 3.1.1. Improvement of the GO Preparation Process

Hummers’ method was commonly used in the laboratory for the preparation of GO due to its short reaction time and high efficiency. However, it has been reported in the literature that KMnO_4_ was prone to generate Mn_2_O_7_ in an environment of concentrated sulphuric acid during the process of preparation, which was prone to exploding under hot stirring. The reaction equation for the generation of Mn_2_O_7_ was as follows [33,61]:

$$KMnO_{4}+3H_{2}SO_{4}\to K^{+}+MnO_{3}^{+}+H_{3}O^{+}+3HSO_{4}^{-}\;MnO_{3}^{+}+MnO_{4}^{-}\to Mn_{2}O_{7}$$

This paper improved the Hummers method by adding a certain amount of water to reduce the concentration of Mn_2_O_7_, which was prone to explosive decomposition, to be generated in the expectation of improving the safety of the preparation process. We compared the volume ratios of different concentrations of H_2_SO_4_ and deionised water using 1 g of graphite powder and 5 g of KMnO_4_ (0.032 mol), and the results of the experiments are shown in Table 3.

From the above table, it can be seen that the colour of the product gradually changes from brownish yellow to black as the amount of water increases, which indicates that the degree of oxidation of GO decreases. Meanwhile, when the amount of water increased, the weight of the system product decreased. When the amount of water was in the range of 4.5–18 mL, the yield was similar to the Hummers method from the literature [62] without added water; its yield decreased significantly when the deionised water exceeded 22.5 mL, which indicates that the increase in the amount of water reduced the oxidative properties of the system. Additionally, when the amount of water was 27 mL, the amount of H_2_O_2_ used to consume KMnO_4_ rose to 6 mL, indicating that more KMnO_4_ remained in the system, which was due to the higher water content of the system, which reduces the acidity and is unfavourable for the reaction.

**Figure 1:**
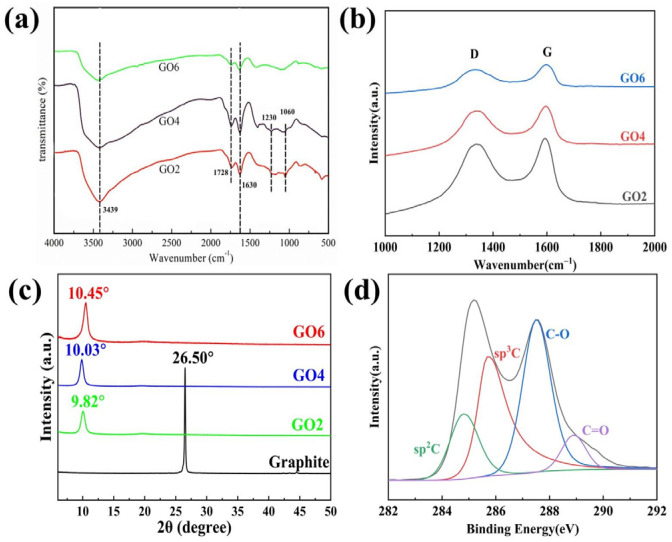
(**a**) IR spectra of GO2, GO4, and GO6; (**b**) Raman spectra of GO2, GO4, and GO6; (**c**) XRD patterns of graphite, GO2, GO4, and GO6; (**d**) XPS spectra of GO4.

We selected GO2, GO4, and GO6 for infrared spectroscopic testing, as shown in Figure 1a. As can be seen from the figure, there are O-H stretching vibrations belonging to hydroxyl and carboxyl groups near 3439 cm^−1^, and there is an absorption peak at 1728 cm^−1^, which is a C=O stretching vibration peak of the carboxyl and carbonyl groups, and there are C-OH stretching vibration peaks and a C-O-C stretching vibration peak near 1230 cm^−1^ and 1060 cm^−1^. This characteristic peak indicates that we prepared GO similar to the conventional Hummers method [62]. 

Figure 1b showed the Raman spectra of GO2, GO4, and GO6. After fitting calculations, the I_D_/I_G_ values of GO2, GO4, and GO6 were 0.88, 0.83, and 0.76, respectively, showing a gradual decrease, which indicates that the oxidation degree decreases with the increase in the amount of water, which was also consistent with the changing colour of the products from bright yellow to black. Compared to the I_D_/I_G_ value of 1.23 for the conventional Hummers method [63], we prepared GO with an overall low value. 

Figure 1c represented the XRD patterns of graphite (Graphite), GO2, GO4, and GO6. From the figure, it can be seen that, after the oxidation of the three GO samples, the sharp characteristic diffraction peak belonging to graphite disappears at 26.5°, and a more clear and sharp diffraction peak appears near 10°, indicating that graphite has been oxidized successfully. The diffraction peak positions 2θ of GO2, GO4, and GO6 are 9.82°, 10.03°, and 10.45°, respectively, and the corresponding layer spacings are 9.00, 8.81, and 8.46 Å. The layer spacing decreases as the oxidation degree decreased. 

We further tested the XPS of GO4 as shown in Figure 1d. The C/O ratio of GO was calculated to be 2.17, compared with the C/O ratio of GO prepared by the conventional Hummers method reported in the literature, which is mostly in the range of 2.14–3 [24,25,28], and the oxidation degree of our prepared GO was within this normal range. 

Sim et al. [64] reported that lower C/O GO films were easier to chemically reduce and show higher electrical conductivity, and from the safety of the experiment and experimental data, we chose the system GO4 for subsequent preparation conditions.

#### 3.1.2. GO Stripping Mechanism and Janus Structure Study

**Figure 2:**
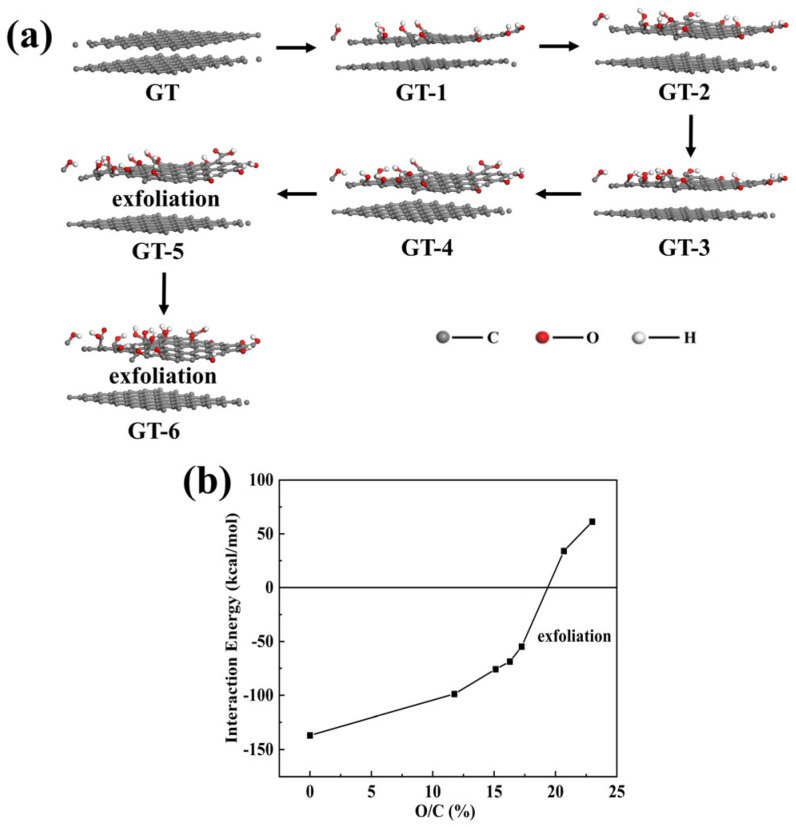
(**a**) Shows the exfoliation process between graphite flakes with different oxidation degrees; (**b**) shows the interaction energy of graphite flakes with different oxidation degrees in H_2_SO_4_.

Dimiev and Tour et al. [65,66] proposed three different independent graphite oxidation processes: the conversion of graphite into a first-stage graphite intercalation compound (GIC), followed by the conversion of the first-stage GIC into pristine graphite oxide (PGO), and lastly, the conversion of PGO into conventional GO after hydrolysis. H_2_SO_4_ has multiple roles in the oxidation process, including acting as an intercalator for the first stage GIC with the assistance of the oxidant, stabilising the oxidant, and acting as a solvent to transport the oxidant between graphite layers. Our analysis suggests that graphite is composed of sp2 with strong interlayer forces, and with the oxidation of the face graphite flakes, the graphite gradually converts from sp2 to sp3 structure, and from planar to three-dimensional structure, which makes its interlayer forces weaken and gradually peel off with the increase in oxidation. We used MS simulations to calculate the oxidative stripping process of graphite (Figure 2a) and calculated the interaction energies of graphite flakes with different oxidisations in H_2_SO_4_, as shown in Figure 2b. From the figure, it can be seen that when the graphite flakes start to oxidise, their overall interaction energy and individual energies are gradually decreasing. And it can be inferred that the interaction between graphite oxide flakes and graphite in H_2_SO_4_ can be 0 kcal/mol when the O/C ratio is approximately 19.37%, at which point the stripping begins. The interaction energy is positive when the O/C ratio is greater than 19.37%, indicating that the interaction energy between the graphite oxide flake and graphite flake is less than that between the graphite oxide flake and H_2_SO_4_, which leads to the exfoliation of the graphite oxide flake from the graphite flake to graphene oxide. According to this oxidation–exfoliation process of hydrophobic graphite as well as the fabricated GO before the exfoliation of graphite flakes will only re-face the layer to appear as one-sided oxidation, and thus the graphene oxide is exfoliated, where one side of the graphene oxide is oxidised more than the other, the highly oxidised side behaves in a hydrophilic manner, and the other in a hydrophobic manner; it has a Janus structure. This structure, when exfoliated into a solution (in polar solvents or water), rapidly turns into a bilayer stacked structure with the hydrophilic surface facing outwards and the hydrophobic and hydrophobic surfaces stacked together facing inwards. The current literature has reported that GO has a distinct sharp peak in the XRD spectrum [30,31,32], which can also further prove our point.

#### 3.1.3. Effect of Different Solutions on janus-GO Layer Spacing

**Figure 3:**
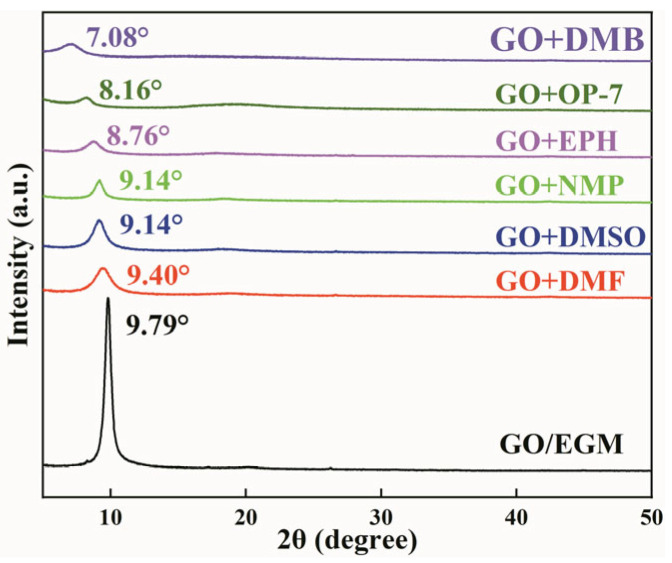
XRD patterns of GO in different solvents.

To further experimentally verify the above theoretical simulation results, we dispersed GO into EGM solution and then added organic solutions with different polarities, such as N,N-dimethylformamide (DMF), dimethylsulfoxide (DMSO), N-methylpyrrolidone (NMP), ethylene glycol phenyl ether (EPH), OP-7, and dimethylbenzene (DMB) for XRD testing of GO, as shown in Figure 3. After the addition of highly polar solvents such as DMF, DMSO, NMP, and EPH to the GO/EGM solution, the XRD peaks were shifted to 9.40°, 9.14°, 9.14°, and 8.76°, with layer spacings of 9.40, 9.66, 9.66, and 10.08 Å, respectively. The addition of weakly polar solvents such as OP-7 and DMB resulted in the shifting of the XRD peaks to 8.16° and 7.08°, and the layer spacing was 10.83 and 12.18 Å. This indicates that weakly polar solvents have a greater effect on the layer spacing.

**Figure 4:**
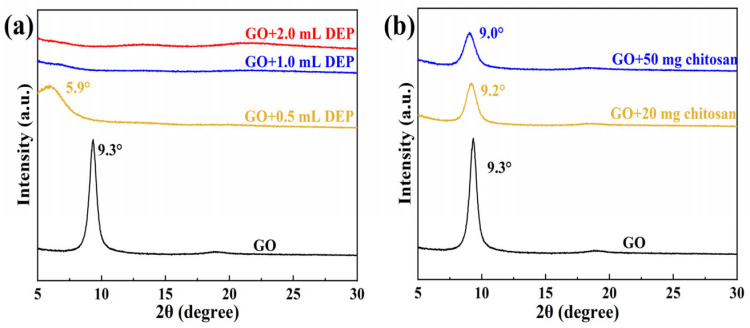
(**a**) XRD patterns of GO incorporating different amounts of DEP; (**b**) XRD patterns of GO incorporating different amounts of chitosan.

In view of the fact that the boiling points of these solvents are relatively low, which can affect the XRD results, we chose the weakly polar diethyl phthalate (DEP) and strongly polar chitosan, which have high boiling points and are not volatile, to be added to the GO/EGM solution for the XRD tests. Figure 4a shows the XRD patterns of GO with 0.5, 1.0, and 2.0 mL of DEP. It can be seen in the figure that a small amount of DEP causes the layer spacing to become larger, but the peak disappears when the addition becomes larger, which indicates that DEP inserts and expands into the GO hydrophobic interlayers, separating the GO layers from each other and forming a monolayer structure. Figure 4b shows the XRD patterns of GO with the addition of 20 mg and 50 mg chitosan. It can be observed that the diffraction peaks of GO basically remained unchanged before and after the addition of hydrophilic chitosan. The diffraction peaks gradually disappeared after the addition of hydrophobic DEP to GO, whereas there was no clear change in the diffraction peaks after the addition of hydrophilic chitosan; these features indicate that GO has a Janus structure and that its poorly oxidised hydrophobic surfaces stack together to form a bilayered stacked structure in the hydrophilic solvent.

By the solvent method, we showed that the diffraction peaks gradually disappeared after the addition of hydrophobic DEP to GO, while there was no obvious change in the diffraction peaks after the addition of hydrophilic chitosan, and these features indicate that GO has a Janus structure with inconsistent oxidation on both sides, and in polar solvents, the hydrophilic water surface with high oxidation faces outward, and the hydrophobic water surface with poor oxidation faces inward to stack with each other and form a double-layer stacked structure. Meanwhile, the oxidation–exfoliation process of graphite was simulated by MS theory, which further proved the Janus structure of GO.

### 3.2. Self-Assembly Studies of janus-GO in Different Solutions

**Figure 5:**
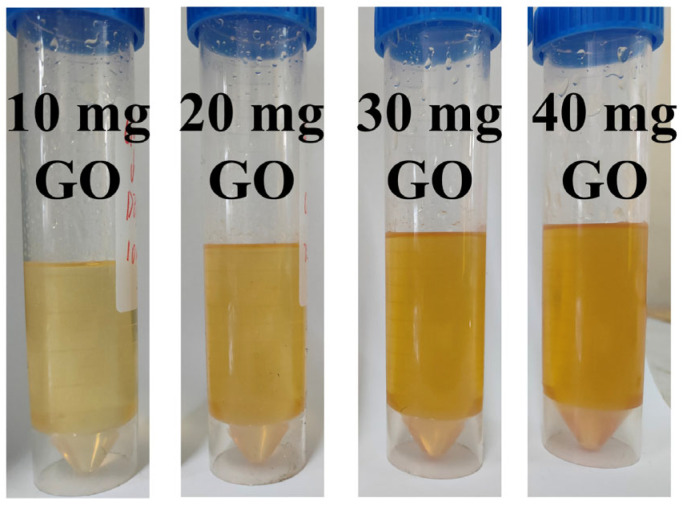
Phenomenon of different contents of GO dispersed into DEP.

It had been reported in the literature that GO is insoluble in weakly polar solutions such as hexane and chloroform [50,51]. Through preliminary experiments, the interlayer spacing disappears when a small amount of DEP is added to the GO/EGM solution, and we believed that DEP inserted and expanded into the GO hydrophobic interlayer. For this reason, we increased the amount of DEP, and dispersed 1.45 mL, 2.85 mL, 4.30 mL, and 5.75 mL of GO/EGM solution (GO contents of 10 mg, 20 mg, 30 mg, and 40 mg, respectively) into 20 mL of DEP, and found that all the solutions became transparent (as in Figure 5), but with the gradual increase in the content of GO, the viscosity of their liquids also increased, indicating that the interlayer structure of GO was altered in DEP, and it was possible that the chain structure was formed by affecting the van der Waals forces and hydrogen bonding between the GO lamellae.

**Figure 6:**
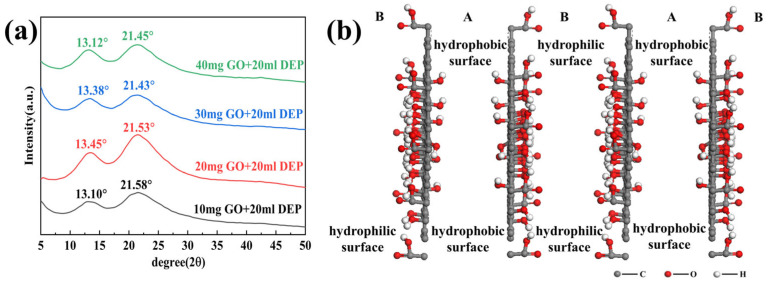
(**a**) XRD patterns of GO/EGM solutions with different amounts added to DEP; (**b**) multilayer structure of GO.

In order to further explore the structural alterations in DEP, we subjected the above samples to XRD tests as shown in Figure 6a. As can be seen from the figure, when the GO/EGM solution was added directly dropwise into DEP, clear double peaks appeared at about 13.5° (A) and 21.5° (B), corresponding to layer spacings of 6.55 Å and 4.13 Å. The change from sharp single peaks to broad diffuse double peaks indicated the formation of a multilayered stacked structure (Figure 6b), which also explains the increase in viscosity, which may be related to the formation of a GO with a continuous chain structure.

**Figure 7:**
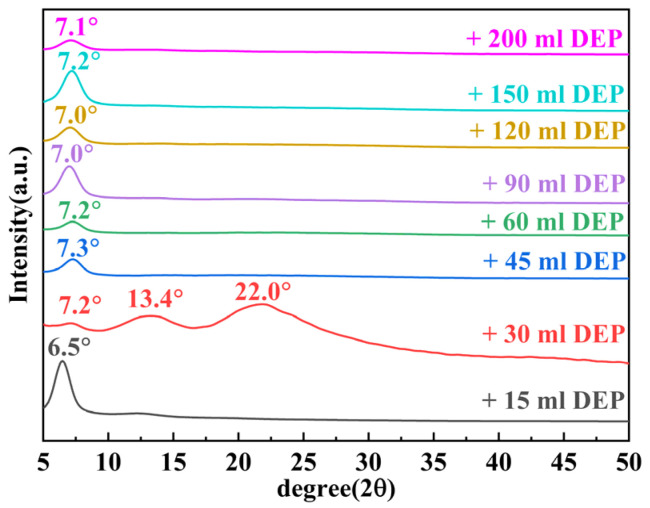
XRD patterns of GO/EGM solution added to benzyl alcohol to which different amounts of DEP were added.

Due to the poor phase solubility between EGM and DEP, we added 5.75 mL of GO/EGM solution (GO content of 40 mg) to 20 mL of benzyl alcohol, and then added different amounts of DEP to it, and performed XRD tests on different mixtures (Figure 7). From the figure, it can be seen that when the content of DEP was 15 mL, it showed a single peak; when the content was 30 mL, the peak became a broad diffuse peak again and a new double peak appeared, which was when GO appeared in a continuous chain structure; when the content was more than 45 mL, it changed back to a single peak at about 7.1° (with the layer spacing of about 12.44 Å). This change may occur because the hydrophobic surface of GO completely changes from an inward-facing stacked structure to a single assembled structure with the hydrophilic surface facing inward and the hydrophobic surface facing outward as the weakly polar content in the mixed solution increases.

To further explain the changes in GO molecular structure in different solvents, we simultaneously calculated the interaction energies of janus-GO in BnOH, DEP, and water, and distinguished between hydrophobic surfaces (A side), where GO is poorly oxidised, and hydrophilic surfaces (B side), where it is highly oxidised. Table 4 represents the interaction energies of the A and B surfaces of janus-GO in BnOH, DEP, and water, respectively. In water, the interaction energy of janus-GO-A-A is −12.94 kcal/mol, indicating that the interaction energy between the two hydrophobic surfaces is greater than that between them and water, and the hydrophobic surfaces of janus-GO tend to stack inward to form a bilayered stacked structure; and the interaction energy of janus-GO-B-B is 75.38 kcal/mol, indicating that the interaction energy between the two amphiphilic surfaces is smaller than that between them and water, and the amphiphilic surfaces of janus-GO tend to be dispersed in the water towards the outside. Conversely, the interaction energies of janus-GO in BnOH and DEP are slightly different from that of water. The interaction energies of the hydrophobic and amphiphilic surfaces of janus-GO in solvent are both positive, but the interaction energies of janus-GO-A-A are higher than those of janus-GO-A-B and Janus B-B, which indicate that janus-GO is mainly stacked together in the form of hydrophobic surface facing outward in BnOH and DEP, which indicates that janus-GO “turns over” in benzyl alcohol and DEP. BnOH and DEP are mainly stacked together in the form of hydrophobic surfaces facing outward and hydrophilic surfaces facing inward, indicating that janus-GO is “flipped” in benzyl alcohol and DEP. This also explains why GO can be well solubilised in BnOH, an oil-soluble reagent with low polarity. Meanwhile, comparing the interactions of janus-GO in benzyl alcohol and DEP, the energy in DEP is lower than that in benzyl alcohol. This is due to the lower polarity of DEP compared to benzyl alcohol, which reduces the bond energy for binding with oxygen-containing functional groups on the GO surface.

### 3.3. Preparation and Structural Characterisation of Conductive Thin Films Based on Benzyl Alcohol Solution

#### 3.3.1. Primary Reduction

**Figure 8:**
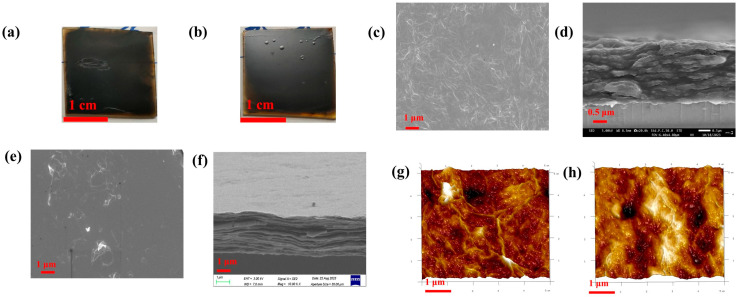
(**a**) Optical photographs of phenylhydrazine-prepared conductive thin films (PHrGO); (**b**) optical photographs of hydrazine hydrate-prepared conductive thin films (rGO); (**c**,**d**) SEM images of PHrGO; (**e**,**f**) SEM images of rGO; (**g**,**h**) AFM images of PHrGO and rGO, respectively.

The preparation of conductive films in aqueous solutions has been reported in the literature [67,68], and in this paper; conductive films were prepared by reduction using a two-phase system of benzyl alcohol and water, which is incompatible with water. By treating one glass surface with KH550 and the other glass surface without any other treatment, the experimental results showed that the glass surface not treated with KH550 could not form a continuous film, while the hydrophobic surface with added KH550 was able to form a film better, which indicates that GO is selective in the two-phase system. Therefore, we used phenylhydrazine and hydrazine hydrate as reducing agents to prepare conductive films on the glass surface treated with KH550; Figure 8a shows the optical photographs of the conductive film (PHrGO) prepared with phenylhydrazine and Figure 8b shows the optical photographs of the conductive film (rGO) prepared with hydrazine hydrate. From the figures, it can be seen that the two films are macroscopically homogeneous and the films were further tested by SEM (Figure 8c–f). The interlayer structure of phenylhydrazine is more sparse as can be seen in Figure 8c,d, whereas the films prepared from hydrazine hydrate in Figure 8e,f have a thinner lamellar structure and have more number of lamellar layers than the films prepared from phenylhydrazine. The surface of the films was further characterised using AFM. In Figure 8g, it can be observed that the film prepared by phenylhydrazine (PHrGO) has a distinct folded stacked structure with higher surface roughness, whereas the film prepared by hydrazine hydrate (rGO) has a lower roughness. The thickness of the films was tested to be 4–20 nm (Figure S1, Supporting Information), i.e., the number of layers was about 4–20. The electrical conductivity of the films prepared by primary reduction with phenylhydrazine was 177 S/m by four-probe method, whereas the electrical conductivity of the films prepared by primary reduction with hydrazine hydrate was 1053 S/m by four-probe method, which may be related to the stronger reducing property of hydrazine hydrate.

#### 3.3.2. Secondary Reduction

##### Conductive Films Prepared with Phenylhydrazine (PHrGO)

**Figure 9:**
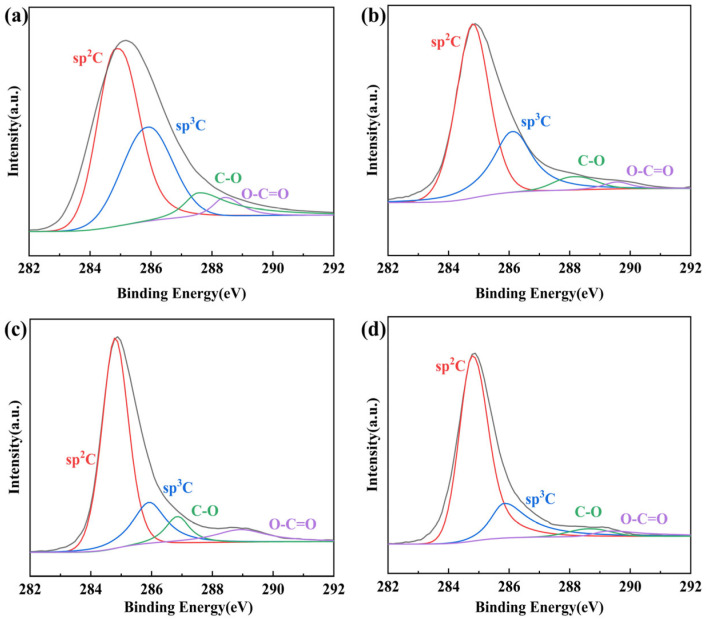
XPS spectra of (**a**) PHrGO; (**b**) PHrGO-1; (**c**) PHrGO-2; (**d**) PHrGO-3.

In order to further improve the conductivity of the films, we used HI for secondary reduction of the films produced by primary reduction. We compared the effects of different amounts of HI, NaI, and deionised water in the compound solution on the reduction effect of the conductive films, respectively. Table 5 and Figure S3 (Supporting Information) shows the conductivity and C/O ratio of PHrGO, PHrGO-1, PHrGO-2, and PHrGO-3. From the table, it can be seen that the combination of HI/NaI/H_2_O has the highest conductivity when used as a reducing agent. Figure 9a–d shows the XPS spectra of PHrGO and PHrGO with different reducing agents for secondary treatment. Observation of the plots shows that both the PHrGO films and its films after secondary reduction have sp2 carbon as the main peak, and the area of the sp3 carbon peak slowly becomes smaller with the increase in the reduction degree.

**Figure 10:**
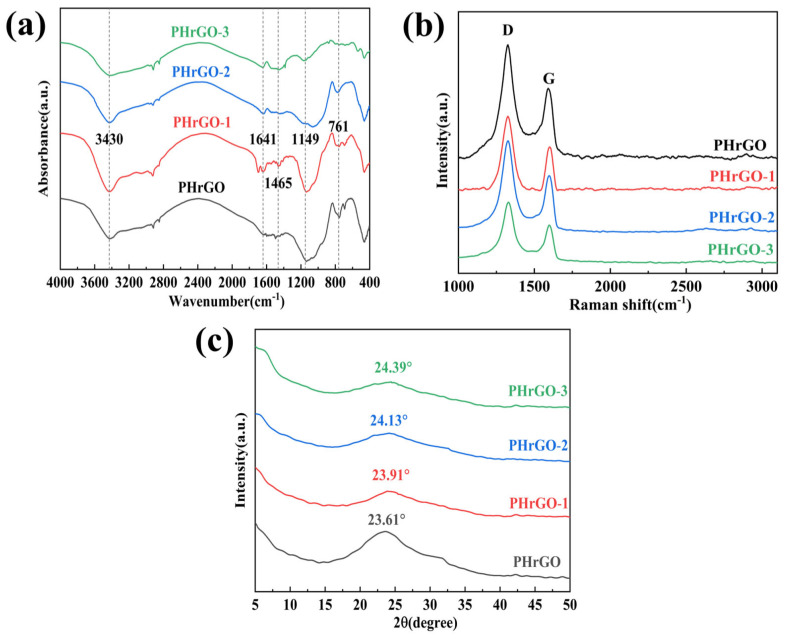
PHrGO, PHrGO-1, PHrGO-2, and PHrGO-3: (**a**) FTIR; (**b**) Raman; (**c**) XRD.

PHrGO, PHrGO-1, PHrGO-2, and PHrGO-3 were characterised using IR spectroscopy as shown in Figure 10a. The O-H telescopic vibrations of hydroxyl and carboxyl groups are around 3430 cm^−1^, the absorption peaks caused by the telescopic vibrations of carbonyl group (C=O) are at 1641 cm^−1^, the absorption peaks caused by the telescopic vibrations of alkoxyl group (C-O) are at 1149 cm^−1^, and the intensities of these vibration peaks decrease with the increase in the reduction degree. The above data indicate that the conductive film was effectively reduced and the oxygen-containing functional groups were eliminated. The results are consistent with the XPS results.

The degree of defects in the reduced graphene oxide products was further characterised by Raman spectroscopy, and the I_D_/I_G_ values in the Raman spectra can reflect the defect density of graphene materials. Figure 10b shows the Raman plots of PHrGO, PHrGO-1, PHrGO-2, and PHrGO-3. Computer fitting was performed to find the I_D_/I_G_ values, as shown in Table 6 below. The intensity ratios between the I_D_ and I_G_ bands of the films prepared from PHrGO prepared by phenylhydrazine and after secondary reduction with different reducing agents are 1.54, 1.50, 1.45, and 1.42, respectively. The higher intensity of the D band as compared to the G band is due to the increase in the disordered phase of the films with the sp3 hybridised bonds formed by the oxidation of graphite after reduction.

PHrGO, PHrGO-1, PHrGO-2, and PHrGO-3 were characterised using XRD as shown in Figure 10c. It can be seen that the 2θ of PHrGO, PHrGO-1, PHrGO-2, and PHrGO-3 are 23.61°, 23.91°, 24.13°, and 24.39°, respectively, and the interlayer spacing is calculated from Bragg’s equation as 3.76 Å, 3.72 Å, 3.68 Å, and 3.65 Å. The interlayer spacing is decreasing with the higher degree of reduction.

##### Conductive Films Prepared with Hydrazine Hydrate (rGO)

**Figure 11:**
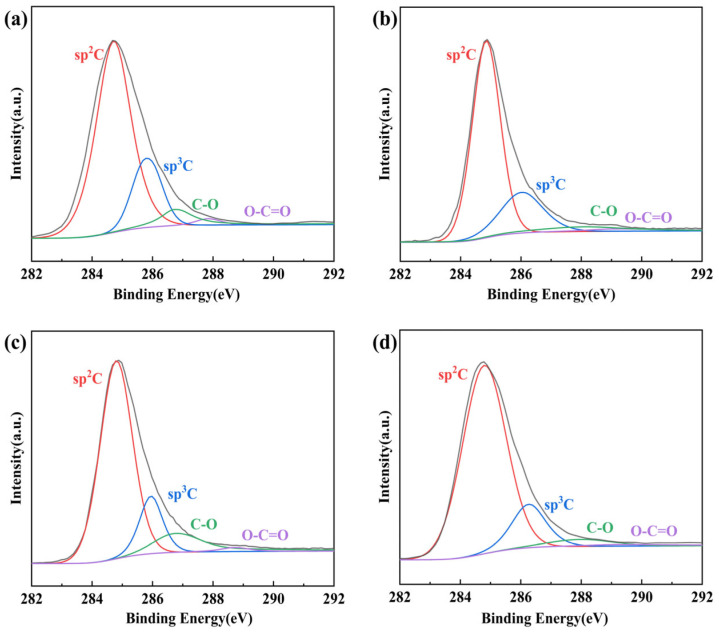
XPS spectra of (**a**) rGO; (**b**) rGO-1; (**c**) rGO-2; (**d**) rGO-3.

From the reduction data of phenylhydrazine, the primary and secondary reduction also did not increase the conductivity of the films too much, so we used the films prepared with the more reducing hydrazine hydrate for the secondary reduction. Table 7 and Figure S2 (Supporting Information) shows the conductivity and C/O ratio of rGO, rGO-1, rGO-2, and rGO-3. From the table, it can be seen that the combination of HI/NaI/H_2_O has the highest conductivity. Figure 11a–d shows the XPS spectra of GO and rGO treated with different reducing agents. From the figure, it can be seen that, although the intensity of the absorption peaks of sp2 C in rGO was enhanced, the intensity of the absorption peaks of C-O and O-C=O groups was still large, indicating that there were still many oxygen-containing functional groups in rGO that were not removed and not reduced completely. Compared with rGO, the absorption peak intensities of sp2 C in rGO-1, rGO-2, and rGO-3 were greatly enhanced, the absorption peak intensities of sp3 C were greatly reduced, the absorption peak intensities of C-O groups were greatly reduced, and the absorption peak intensities of O-C=O groups were already quite weak and basically disappeared, and it was obvious that rGO-3 was reduced more completely.

**Figure 12:**
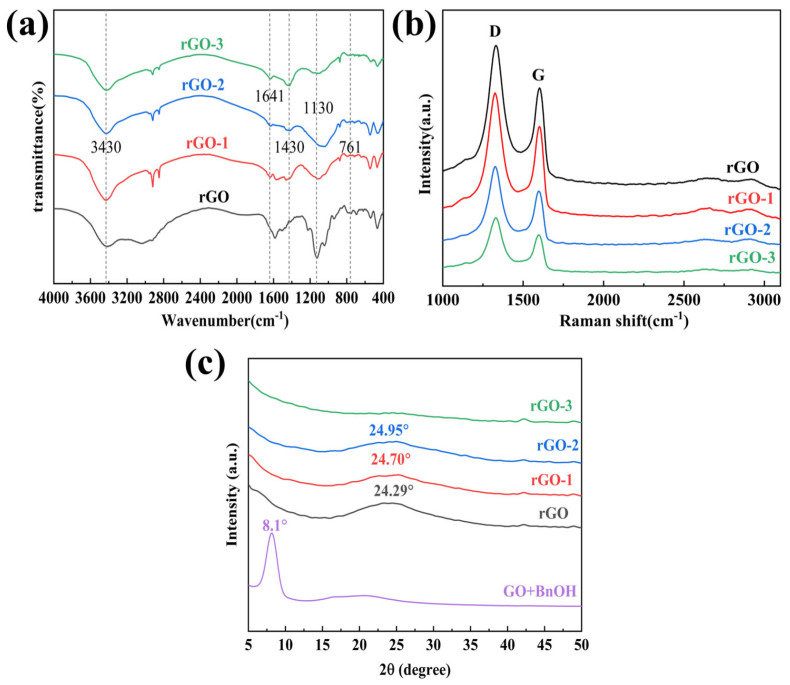
rGO, rGO-1, rGO-2, and rGO-3: (**a**) FTIR; (**b**) Raman; (**c**) XRD patterns of benzyl alcohol solution of GO and of rGO, rGO-1, rGO-2, and rGO-3.

The rGO, rGO-1, rGO-2, and rGO-3 were characterised using IR spectroscopy as shown in Figure 12a. The O-H stretching vibrations belonging to hydroxyl and carboxyl groups near 3430 cm^−1^, the absorption peaks caused by the stretching vibration of carbonyl group (C=O) at 1641 cm^−1^, and the absorption peaks caused by the stretching vibration of alkoxy group (C-O) at 1130 cm^−1^, which decrease in the intensity of the vibration peaks with the increase in the reduction degree. The above data indicate that the conductive film was effectively reduced and all oxygen-containing functional groups were eliminated. Meanwhile, as can be seen from the figure, the absorption peaks are caused by the bending vibration of O-H at 1430 cm^−1^; rGO-3 is almost absent, which can be seen from the fact that the reduction degree is the highest. The results are consistent with the XPS results.

The defect densities of graphene oxide and graphene were characterised by Raman, and usually a larger value of I_D_/I_G_ indicates a higher defect density. rGO, rGO-1, rGO-2, and rGO-3 Raman plots, as shown in Figure 12b. Computer fitting was performed to find the I_D_/I_G_ values, as shown in Table 8 below. From the table, it can be seen that the I_D_/I_G_ values of rGO after reduction are all larger than that of GO, which is due to the fact that the oxygen-containing functional groups on the surface of GO are removed when GO is reduced, and a large number of sp3 structures are changed into sp2 structures during the reduction process, and the degree of graphitization is increased. The I_D_/I_G_ of rGO-3, on the other hand, is 1.31, which has the lowest defect density among the rGO series, which proves that it possesses more sp2 structures and the highest electrical conductivity.

XRD tests were performed on GO/BnOH solution, rGO, rGO-1, rGO-2, and rGO-3 as in Figure 12c. By comparing Figure 12c with Figure 1c, it can be seen that the peak position of GO/BnOH solution is at 8.1°, and its layer spacing is calculated to be 10.9 Å, which is larger than that of GO4 (8.81 Å), implying that the incorporation of BnOH, which acts as some spatial site barrier, opens up the layer spacing of GO, and effectively disrupts the GO structure. From Figure 12c, it can be seen that at 24.29°–24.95° the broad diffraction peaks appear, which means that they are dispersed more openly from layer to layer. Meanwhile, the intensities of the peaks are all lower, indicating that the increase in the pre-existing layer spacing is conducive to the reaction of the reductant as much as possible with the GO between the two lamellae, which improves the degree of reduction and the conductivity increases.

#### 3.3.3. Reduced Graphene Oxide Patterns

**Figure 13:**
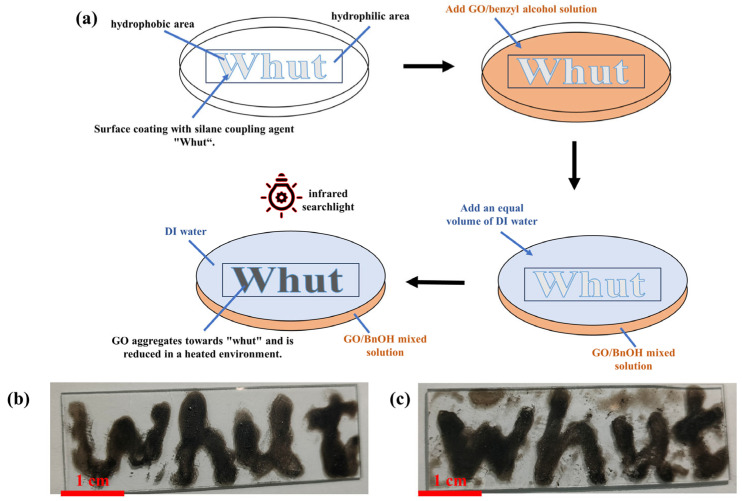
(**a**) Flowchart of reduced graphene oxide patterns; (**b**,**c**) optical photographs of “Whut” patterns prepared using phenylhydrazine and hydrazine hydrate as reducing agents, respectively.

Based on the results of the above study, we tried to prepare thin film patterns for the study. The hydrophobic pattern of “Whut” was coated on the substrate with silane coupling agent (KH550), and a slightly water-soluble BnOH solution containing GO was added to cover the glass surface, and then an equal volume of water with benzyl alcohol was added to make use of water’s affinity for polar substrates, so that the benzyl alcohol solution of GO was enriched in the hydrophobic pattern area through the exclusion of benzyl alcohol, and then the reduction was accelerated by infrared lamp irradiation, and finally the “Whut” pattern was obtained. By squeezing benzyl alcohol, the benzyl alcohol solution of GO is enriched in the hydrophobic pattern area, and then the reduction is accelerated by infrared lamp irradiation to obtain the “Whut” pattern, which is shown in Figure 13a. Here, two reducing agents, phenylhydrazine and hydrazine hydrate, were used for the study, one of which dissolved phenylhydrazine in the oil phase of the GO benzyl alcohol solution; the other dissolved hydrazine hydrate in the water phase, and the resulting patterns are shown in Figure 13b,c. It is clear that the pattern using phenylhydrazine as a reducing agent has better clarity, which is due to the fact that its reducing agent, phenylhydrazine, is in the oil phase, whereas it can be reduced directly in the oil phase.

**Figure 14:**
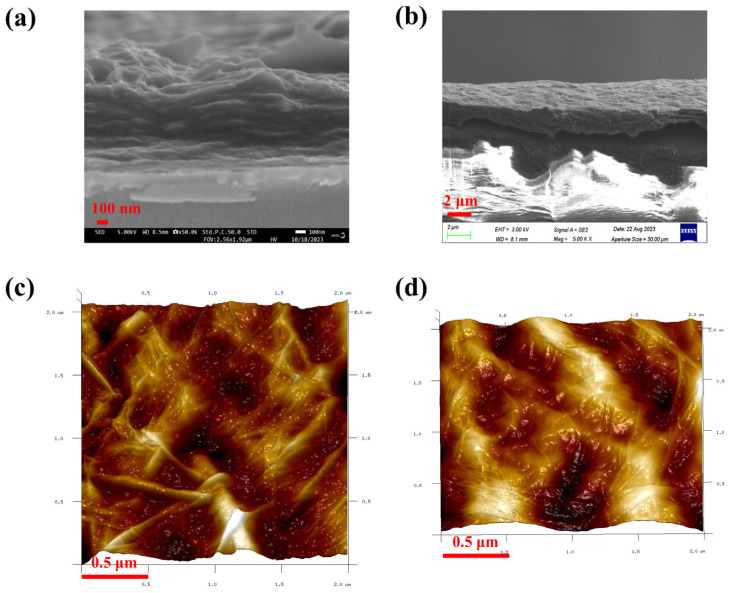
(**a**,**b**) SEM images of patterns prepared using phenylhydrazine and hydrazine hydrate as reducing agents, respectively; (**c**,**d**) AFM images of patterns prepared using phenylhydrazine and hydrazine hydrate as reducing agents, respectively.

The “Whut” patterns prepared using phenylhydrazine and hydrazine hydrate as the reducing agents were subjected to SEM and AFM tests, as shown in Figure 14a–d. From Figure 14a,c, it can be seen that the patterns prepared with phenylhydrazine are looser and have more grooves between the layers, but they are more tightly fitted on the glass surface. While in Figure 14b,d, using hydrazine hydrate as a reducing agent, the lamellae are more tightly packed, but the fit on the glass surface is not as good.

## 4. Conclusions

We effectively reduced the generation concentration of Mn_2_O_7_, which is prone to explosive decomposition, in GO prepared by the Hummers method by adding a certain amount of water. We proved that GO is a Janus structure with inconsistent oxidation on both sides by mixed solvent method and theoretical simulation. Meanwhile, we found that the change in solution polarity causes the layer spacing to change and the lamellar structure to flip. Based on the stacked structure of GO in benzyl alcohol, we used a two-phase system of benzyl alcohol and water, as well as by controlling the polarity of the substrate surface, a mixed solution of GO has a selectivity that is more inclined to be lipophilic when it is present in the aqueous phase, to achieve (using a mixed solution of GO which has a selectivity more inclined to the oil phase when the aqueous phase is present) the preparation of reduced graphene oxide patterns. Meanwhile, the conductivity was enhanced to 8653 S/m by secondary reduction with a complex solution of HI, NaI, and deionised water.

## Figures and Tables

**Table 1:** Ratio of concentrated H_2_SO_4_ and DI water.

	GO1	GO2	GO3	GO4	GO5	GO6
H_2_SO_4_ (mL)	85.5	81	76.5	72	67.5	63
H_2_O (mL)	4.5	9	13.5	18	22.5	27

**Table 2:** Types and amounts of reducing agents used.

Film Type		HI (mL)	NaI (g)	H_2_O (mL)
Films prepared with phenylhydrazine	PHrGO	-	-	
	PHrGO-1	10	-	
	PHrGO-2	10	2.5	
	PHrGO-3	10	10	
Films prepared with hydrazine hydrate	rGO	-	-	-
	rGO-1	10	-	-
	rGO-2	10	2.5	-
	rGO-3	10	10	5

**Table 3:** Contents of GO preparation for different concentrated H_2_SO_4_–water systems.

	GO1	GO2	GO3	GO4	GO5	GO6
Graphite (g)	1	1	1	1	1	1
KMnO_4_ (mol)	0.032	0.032	0.032	0.032	0.032	0.032
H_2_O (mL)	4.5	9	13.5	18	22.5	27
H_2_SO_4_ (mL)	85.5	81	76.5	72	67.5	63
H_2_O_2_ (mL)	2	2	2	3	3	6
Product weight (g)	1.05	1.01	0.99	0.92	<0.84	<0.5
Product colour	light brown	yellow	yellow	yellow	light brown	black

**Table 4:** Interaction energies of A and B faces of janus-GO in BnOH, DEP, and water.

Solvent	janus-GO	Total Energy (kcal/mol)	Single Energy-A (kcal/mol)	Single Energy-B (kcal/mol)	Interaction Energy (kcal/mol)
H_2_O	janus-GO-A-A	3006.27	1509.61	-	−12.94
	janus-GO-A-B	2379.87	1509.61	829.37	40.89
	janus-GO-B-B	1734.13	-	829.37	75.38
BnOH	janus-GO-A-A	2198.52	1078.21	-	42.11
	janus-GO-A-B	1509.65	1078.21	401.65	29.80
	janus-GO-B-B	816.41	-	401.65	13.11
DEP	janus-GO-A-A	1967.25	969.09	-	29.07
	janus-GO-A-B	1309.35	969.09	329.76	10.50
	janus-GO-B-B	660.33	-	329.76	0.81

**Table 5:** C/O ratio and electrical conductivity of PHrGO, PHrGO-1, PHrGO-2, and PHrGO-3.

	Number of Film Reductions	HI (mL)	NaI (g)	H_2_O (mL)	C/O Ratio (%)	Electrical Conductivity (S/m)
PHrGO	Primary reduction	-	-	-	5.04	177
PHrGO-1	Secondary reduction	10	-	-	6.11	458
PHrGO-2	Secondary reduction	10	2.5	-	6.99	989
PHrGO-3	Secondary reduction	10	10	5	7.59	2579

**Table 6:** Raman spectroscopy of PHrGO, PHrGO-1, PHrGO-2, and PHrGO-3.

	D Band (cm^−1^)	G Band (cm^−1^)	I_D_/I_G_ (%)
PHrGO	1324	1589	1.54
PHrGO-1	1324	1599	1.50
PHrGO-2	1327	1591	1.45
PHrGO-3	1324	1591	1.42

**Table 7:** C/O ratio and electrical conductivity of rGO, rGO-1, rGO-2, rGO-3.

	Number of Film Reductions	HI (mL)	NaI (g)	H_2_O (mL)	C/O Ratio (%)	Electrical Conductivity (S/m)
rGO	Primary reduction	-	-	-	7.49	1053
rGO-1	Secondary reduction	10	-	-	8.48	2539
rGO-2	Secondary reduction	10	2.5	-	8.96	5570
rGO-3	Secondary reduction	10	10	5	9.47	8653

**Table 8:** Raman spectroscopy of GO, rGO, rGO-1, rGO-2, and rGO-3.

	D Band (cm^−1^)	G Band (cm^−1^)	I_D_/I_G_ (%)
rGO	1330	1592	1.40
rGO-1	1327	1590	1.39
rGO-2	1324	1591	1.37
rGO-3	1325	1592	1.31

## Data Availability

Data are contained within the article and Supplementary Materials.

## Supplementary Materials

The following supporting information can be downloaded at: https://www.mdpi.com/article/10.3390/nano14110980/s1, Figure S1. AFM images of reduced graphene oxide films. Figure S2. Image of conductivity testing of rGO film using a four-probe tester. Figure S3. Image of conductivity testing of PHrGO film using a four-probe tester. Figure S4. Optical photograph of a multi-function digital four probe tester. Table S1. Parameters of Multi-Function Digital Four-Probe Tester.
