# Supplementary material for: The Janus Structure of Graphene Oxide and Its Large-Size Conductive Film Strip Pattern
Source: Nanomaterials (Basel). 2024 Jun 5;14(11):980. doi: 10.3390/nano14110980 (PMC11173957; doi:10.3390/nano14110980)
Supplement: Supplementary file 1 [file nanomaterials-14-00980-s001.zip › nanomaterials-3017197-supplementary.pdf]

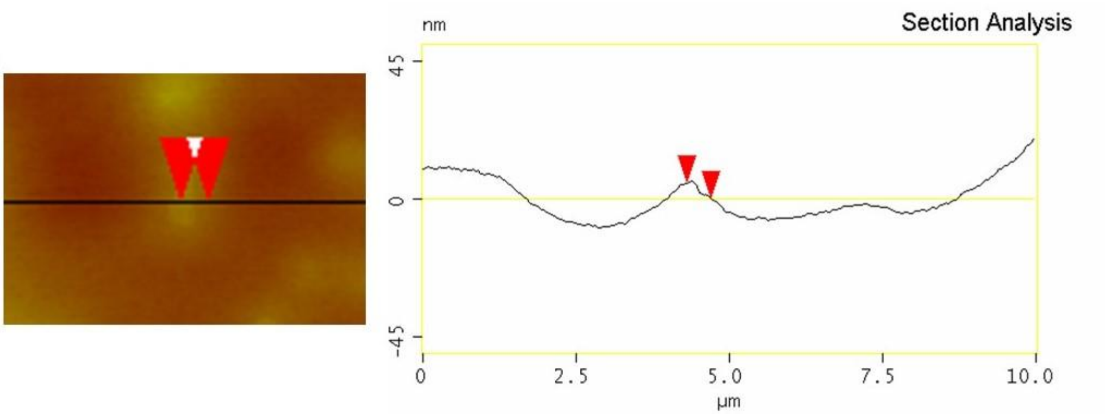


**Figure S1.** AFM images of reduced graphene oxide films**.**

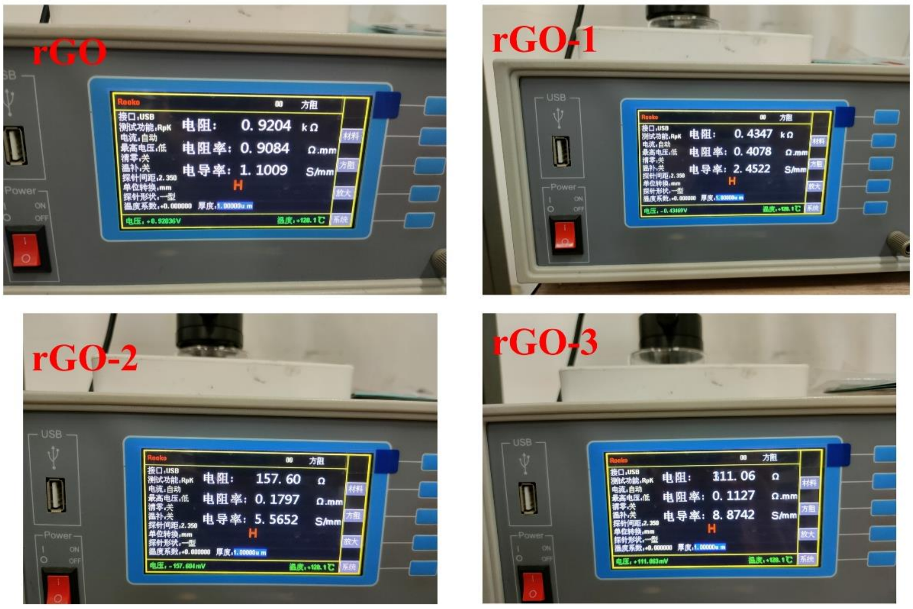


**Figure S2.** Image of conductivity testing of rGO film using a four probe tester

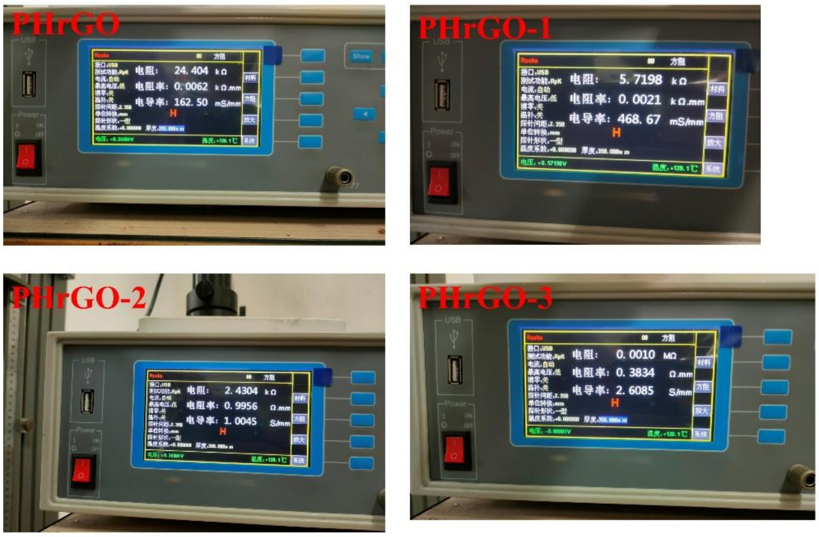


**Figure S3.** Image of conductivity testing of PHrGO film using a four probe tester

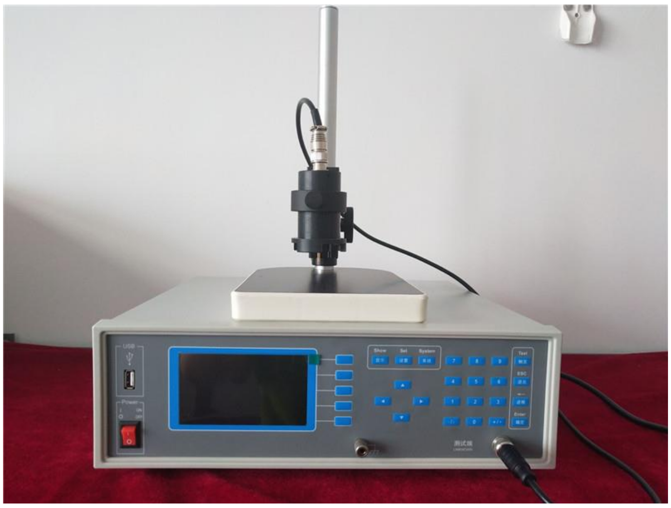

**Figure S4.** Optical photograph of a multi-function digital four probe tester.

**Table S1.** Parameters of Multi-function Digital Four Probe Tester

| items | parametric |
|---|---|
| Square Resistance Range | $10^{-5}$～$2\times10^{5}$ Ω/sq |
| Resistivity range | $10^{-5}$～$2\times10^{6}$ Ω • cm |
| Test current range | 0.1μA， 1μA， 10μA， 100μA， 1mA， 10mA， 100 mA |
| Current Accuracy | ± 0.1% |
| Resistance Accuracy | ≤ 0.3% |
| Display readout LCD | Resistance, resistivity, square resistance, temperature, unit conversion, temperature coefficient, current, voltage, probe shape, probe spacing, thickness, conductivity |
| Test Method | bielectricity measurement |
| Operating power | Input: AC 220V±10%， 50Hz Power Consumption： <30W |
| Uncertainty error of the whole machine | ≤4%（Standard Sample Results） |
| Test Probes | Probe spacing： 2.35mm; |
